# Immortalization capacity of HPV types is inversely related to chromosomal instability

**DOI:** 10.18632/oncotarget.8058

**Published:** 2016-03-14

**Authors:** Denise M. Schütze, Oscar Krijgsman, Peter J.F. Snijders, Bauke Ylstra, Joachim Weischenfeldt, Balca R. Mardin, Adrian M. Stütz, Jan O. Korbel, Johan P. de Winter, Chris J.L.M. Meijer, Wim G.V. Quint, Leontien Bosch, Saskia M. Wilting, Renske D.M. Steenbergen

**Affiliations:** ^1^ Department of Pathology, VU University Medical Center, Amsterdam, The Netherlands; ^2^ Department of Molecular Oncology, The Netherlands Cancer Institute, Amsterdam, The Netherlands; ^3^ Genome Biology Unit, European Molecular Biology Laboratory, Heidelberg, Germany; ^4^ Department of Clinical Genetics, VU University Medical Center, Amsterdam, The Netherlands; ^5^ DDL Diagnostic Laboratory, Voorburg, The Netherlands

**Keywords:** arrayCGH, high-risk HPV, E6/E7, transformation, chromothripsis

## Abstract

High-risk human papillomavirus (hrHPV) types induce immortalization of primary human epithelial cells. Previously we demonstrated that immortalization of human foreskin keratinocytes (HFKs) is HPV type dependent, as reflected by the presence or absence of a crisis period before reaching immortality. This study determined how the immortalization capacity of ten hrHPV types relates to DNA damage induction and overall genomic instability in HFKs.

Twenty five cell cultures obtained by transduction of ten hrHPV types (i.e. HPV16/18/31/33/35/45/51/59/66/70 E6E7) in two or three HFK donors each were studied.

All hrHPV-transduced HFKs showed an increased number of double strand DNA breaks compared to controls, without exhibiting significant differences between types. However, immortal descendants of HPV-transduced HFKs that underwent a prior crisis period (HPV45/51/59/66/70-transduced HFKs) showed significantly more chromosomal aberrations compared to those without crisis (HPV16/18/31/33/35-transduced HFKs). Notably, the hTERT locus at 5p was exclusively gained in cells with a history of crisis and coincided with increased expression. Chromothripsis was detected in one cell line in which multiple rearrangements within chromosome 8 resulted in a gain of MYC.

Together we demonstrated that upon HPV-induced immortalization, the number of chromosomal aberrations is inversely related to the viral immortalization capacity. We propose that hrHPV types with reduced immortalization capacity *in vitro*, reflected by a crisis period, require more genetic host cell aberrations to facilitate immortalization than types that can immortalize without crisis. This may in part explain the observed differences in HPV-type prevalence in cervical cancers and emphasizes that changes in the host cell genome contribute to HPV-induced carcinogenesis.

## INTRODUCTION

A persistent infection with certain types of the human papillomavirus (HPV) has been causally associated with the development of cervical cancer, as well as a subset of other anogenital and head-and-neck cancers. Based on epidemiological data and functionally relevant genomic sequence differences, HPV types of the alpha-genus are classified as high-risk (hrHPV) (i.e. HPV 16, 18, 31, 33, 35, 39, 45, 51, 52, 56, 58 and 59: IARC group 1, frequently found in cervical cancers), probable/possible hrHPV (i.e. HPV 26, 53, 66, 67, 68, 70, 73 and 82: IARC group 2A/B, infrequently found in cervical cancers) and low-risk HPV (e.g. HPV6 and 11, associated with low-grade cervical lesions or condyloma) [1]. The oncogenic potential of the hrHPV types as well as possible and probable hrHPV types (hereafter collectively referred to as hrHPV) primarily resides in two viral genes, E6 and E7, including their regulatory elements and splice sites, that function to amongst others degrade p53 and pRb, respectively [2]. Generally, HPV infections are transient and self-limiting, but persistent infections with deregulated expression of E6 and E7, so-called transforming infections, can drive epigenetic and genetic aberrations and promote malignant progression [2].

High-risk HPV types can induce immortalization of primary keratinocytes *in vitro* via a two-step process, that involves the bypass of two mortality phases (M1 and M2) (reviewed in [3]). M1, or senescence, can be overcome by hrHPV itself, resulting in an extended though still limited lifespan. In this phase hrHPV E6 and E7 induce DNA double strand breaks that are prevented from repair by virus-mediated impairment of cell cycle control [4, 5]. The accumulation of DNA double strand breaks as well as continued telomere shortening during extended cell division may result in a phase of extensive and lethal chromosomal instability, termed the second proliferative lifespan barrier (M2) or crisis period. Subsequent immortalization has been linked to (epi)genetic hits in host cell genes that give a pro-survival phenotype and the activation of the telomere lengthening enzyme telomerase via upregulation of its catalytic subunit hTERT [6, 7]. Studies on (epi)genetic host cell aberrations associated with hrHPV-mediated immortalization have mostly focused on HPV16 and HPV18 [8–11]. In these studies *in vitro* immortalization has been associated with chromosomal aberrations that largely overlap with those described in cervical carcinomas and its high-grade precursor lesions (cervical intraepithelial neoplasia grade 3; CIN3), such as gains at 1q and 3q (reviewed by [12, 13]).

Despite the fact that HPV16 and HPV18 are the most common HPV types detected in cervical cancer, one third of cervical cancers can be attributed to other hrHPV types. To determine whether *in vitro* immortalization capacities and associated host cell aberrations differ amongst the various hrHPV types, we have recently established a large series of HPV-type specific *in vitro* models. Primary human foreskin keratinocytes (HFKs) isolated from two to three independent donors were transduced with the viral oncogenes E6 and E7 of HPV16, 18, 31, 33, 35, 45, 51, 52, 59, 66 and 70 [14]. When studying the immortalization capacities of the different HPV-types, two main groups were distinguished based on their growth behavior in culture. One group, including HPV types 16, 18, 31, 33 and 35, showed the highest growth promoting activities and these HPV-transduced HFKs displayed a continuous growth in culture without an apparent crisis period. The second group consisted of HPV45, 51, 52, 59, 66 and 70 that efficiently induced an extended lifespan, but these HPV transduced HFKs encountered a severe crisis period, a period with strongly reduced cellular growth and widespread cell death. In the cases of HPV45 and HPV51 only one out of three transduced donors, and in the case of HPV59 two out of three donors became immortal. None of the HPV52-transduced HFKs reached immortality, and this type was therefore not included in present study. These data showed that the immortalization capacities vary between hrHPV types, which may reflect a differential dependence on (epi)genetic aberrations in host cell genes. Previously, we did not observe a clear relationship between DNA methylation of several tumor suppressor gene promoters, and growth behavior [15].

This study aimed to determine whether HPV-type dependent immortalization capacities were related to differential DNA damage induction and copy number aberrations in immortal descendants. To this end, we analyzed HPV16-, 18-, 31-, 33-, 35-, 45-, 51-, 59-, 66- and 70-transduced HFKs, from two-to-three donors each, at distinct stages during immortalization, i.e. early passages representing pre-immortal cells in their extended lifespan and later passages representing immortal cell lines. Immortalization was previously determined by detection of telomerase activity and upregulated hTERT expression [14]. In case of HPV45-, 51-, 59-, 66- and 70-transduced cells these are the cells that escaped from crisis. Cells were analyzed for DNA damage using γ-H2AX staining, and DNA copy number aberrations using array comparative genomic hybridization (arrayCGH).

## RESULTS

### Different HrHPV types equally induce DNA double strand breaks

**Figure 1:**
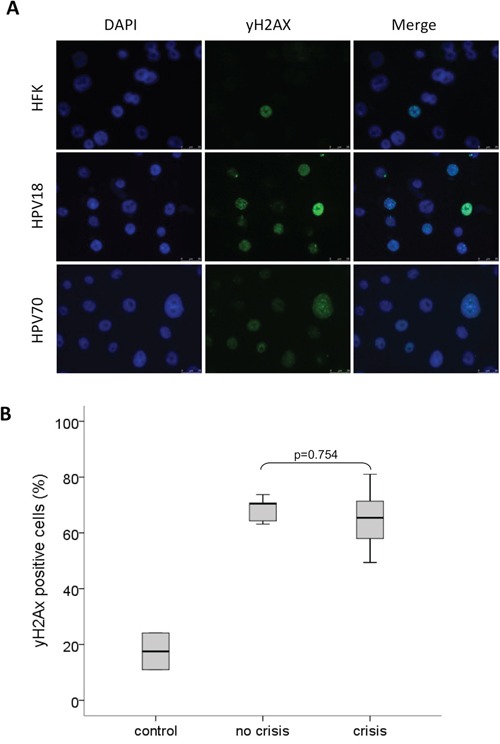
HPV11, 16, 18, 31, 33, 35, 45, 51, 59, 66 and 70 E6E7 induced DNA-double strand breaks **A.** Representative stainings of the nuclei (DAPI) and DNA-double strand breaks (γ-H2A.X) of donor cells (HFK), HPV18- (without crisis) and HPV70- (with crisis) containing HFKs. **B.** Boxplot of γ-H2A.X positive cells (percentage) in controls (including HFKs and lrHPV11), pre-immortal cell lines that did not encounter a crisis prior to immortalization (no crisis, including HPV16, 18, 31, 33 and HPV35) and pre-immortal cell lineages with a crisis period (including HPV45, 51, 59, 66 and 70). The upper and lower boundaries of the boxes represent the 75^th^ and 25^th^ percentiles, respectively. The black line within the box represents the median, the whiskers represent the minimum and maximum values that lie within 1.5 inter quartile range from the end of the box. The experiment was performed in duplicate.

The induction of DNA damage is a well-established feature of hrHPV types such as HPV16 and is known to contribute to HPV-induced cellular transformation (reviewed by [16]). Hence, the observed differential immortalization capacities of various hrHPV types in our established cell cultures of HPV-transduced HFKs may be explained by a type-dependent difference in the extent of DNA damage induction. To study this hypothesis early passages of HPV16-, 18-, 31-, 33-, 35-, 45-, 51-, 59-, 66- and 70-transduced HFKs established in the same donor cells (donor I) were analyzed for the presence of phospho-histone H2A.X (γ-H2A.X), a marker for DNA-double strand breaks (DSBs). Untransduced HFKs and low-risk HPV11 transduced HFKs were included as a control. Control cells showed low γ-H2A.X positive staining (average=17.5%; range 7.7-26.3%), whereas on average 65.8% (range 42.8-89.3%) of hrHPV-transduced cells stained positive for γ-H2A.X (Figure 1A, 1B). Comparison of the number of γ-H2A.X positive cells between cultures showing continuous growth (HPV16-, 18-, 31-, 33- and 35-transduced HFKs) and cultures encountering a crisis period (HPV45-, 51-, 59-, 66- and 70-transduced HFKs) upon immortalization revealed no significant differences (p= 0.754) suggesting that differences in growth behavior between HFKs transduced with the various HPV-types are not associated with differential induction of DNA double-strand breaks.

### Immortal descendants that underwent a crisis period have significantly more DNA copy number aberrations (CNA)

**Figure 2:**
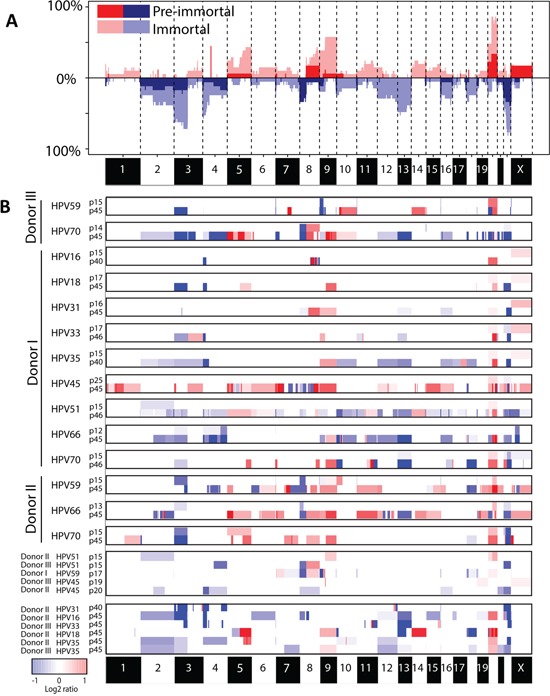
Chromosomal profiles in pre-immortal and immortal cell lines **A.** Frequency plot of pre-immortal cell lines (dark red and blue) (n=19) and immortal cell lines (light red and blue) (n=20). **B.** Matched pre-immortal and immortal passages of the individual cell lines were shown followed by cell lines of which only one passage was present. Red indicates a gain and blue a loss.

**Figure 3:**
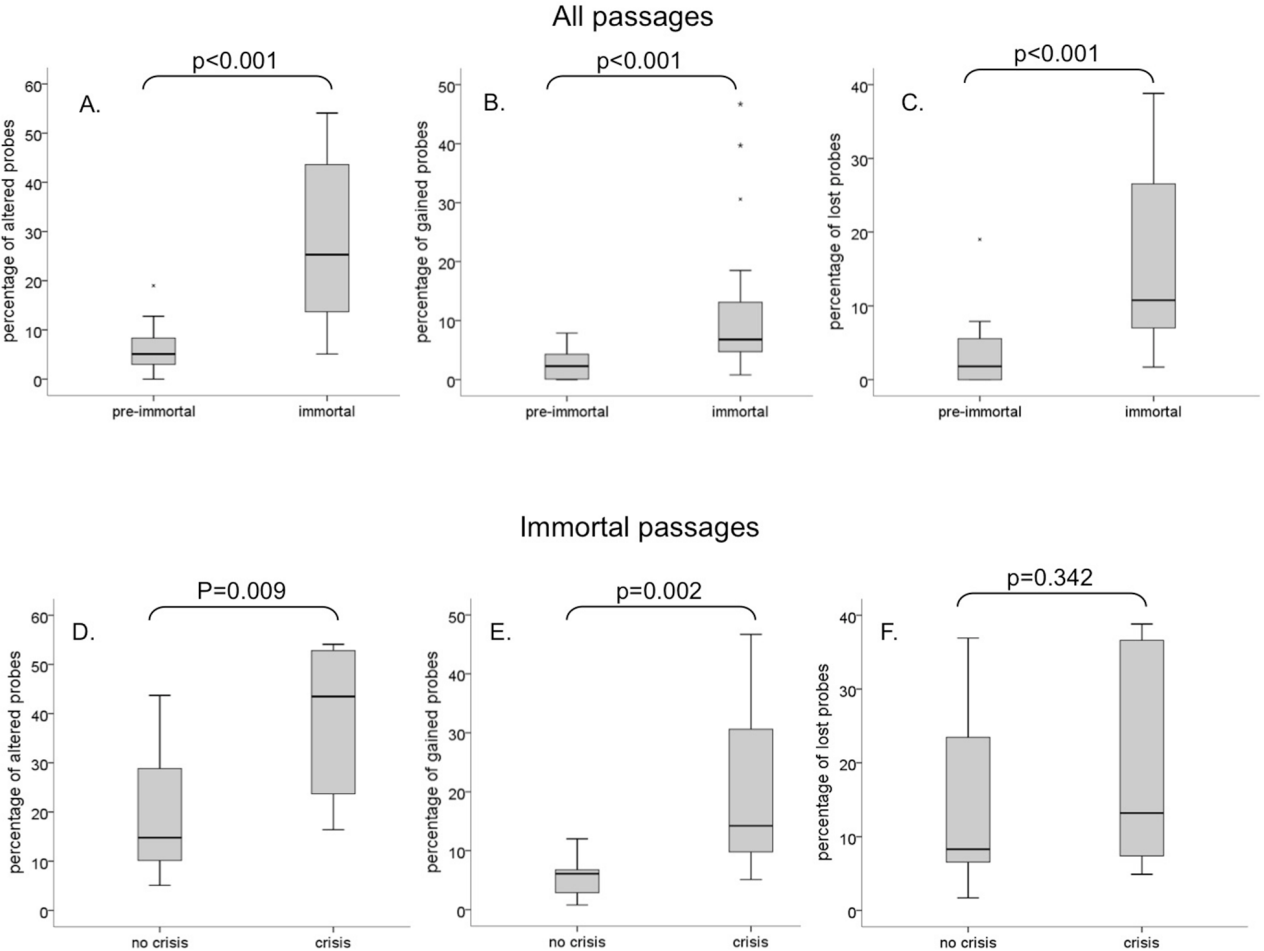
Boxplots showing the percentage of total aberrations, gains and losses Pre-immortal and immortal cells are compared for **A.** percentage of total aberrations, **B.** percentage of gains and **C.** percentage of losses. Cells with and without crisis were compared for percentage of total aberrations, gains and losses in immortal **D-F.** passages. The upper and lower boundaries of the boxes represent the 75^th^ and 25^th^ percentiles, respectively. The black line within the box represents the median, the whiskers represent the minimum and maximum values that lie within 1.5 inter quartile range from the end of the box.

Illegitimate repair of DSBs can lead to CNAs, which could explain the observed differences in growth behavior between the HPV types. We therefore compared the genome-wide chromosomal copy number aberrations observed in pre-immortal and immortal passages (Figure 2A, 2B). A total of 19 pre-immortal and 20 immortal cell cultures (donor I-III, transduced with E6 and E7 from HPV16, 18, 31, 33, 35, 45, 51, 59, 66 and/or 70; see Supplementary Table 1) were analyzed on a 105K arrayCGH platform. The CNAs observed in pre-immortal cells were conserved in their corresponding immortal descendants, which had accumulated more alterations. To enable a comparison of the extent of CNA among different cell lines we determined the percentage of probes on the array deviating from the normal state, hereafter referred to as the percentage of aberrations. Immortal cells showed significantly higher percentages of both gains and losses of copy numbers as compared to pre-immortal passages (p<0.001) (Figure 3A-3C).

In pre-immortal passages, no difference was observed in the percentage of aberrations between cell lines that were to undergo crisis before immortalization and cell lines that were to immortalize without an apparent crisis period (data not shown). However, upon comparison between immortal cells that emerged from continuous growing ancestors and immortal cells that escaped from crisis, significantly more CNA were detected in the post-crisis cells (Figure 3D), which was mainly attributable to a strong increase in the percentage of gains (Figure 3E), rather than losses (Figure 3F).

### Regions on chromosome 5p, 8 and 9q are specifically altered in immortal cells that underwent crisis

**Figure 4:**
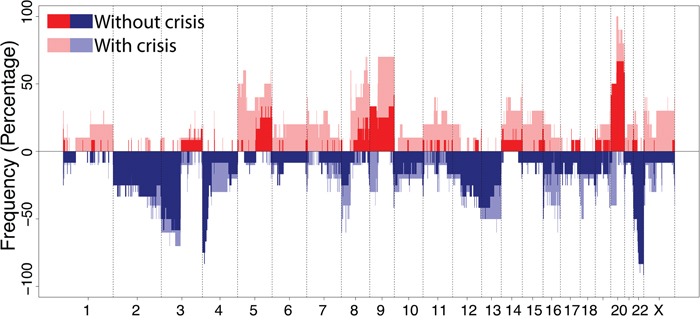
Frequency plot of DNA copy number aberrations in immortal cell lines Cell lineages without crisis including HPV16, 18, 31, 33 and 35 (n=11) are shown in dark red and blue, and cell lines with a crisis prior to immortalization including HPV45, 51, 59, 66 and 70 (n=9) are shown in light red and blue. Red indicates a gain and blue a loss.

A frequency plot for immortal cell lines with (n=9) and without (n=11) a preceding crisis period is shown in Figure 4. Overall, gains of regions on chromosome 20q and losses at chromosome 3p and 22q were observed in more than 70% of cell lines. An overview of all smallest regions of overlap occurring in at least 30% of cell lines is summarized in Table 1.

**Table 1:** Smallest regions of overlap found in at least 30% of all immortal descendants analyzed

GAINS									
Chromosome	Start position	End position	Cytoband	Samples (n=)	Crisis (n=)	No crisis (n=)	% Total	% Crisis	% No crisis
5	33485408	45972937	5p13.3-p11	6	6	0	30.00%	66.67%	0.00%
5	124833923	139945983	5q23.2-q31.3	8	5	3	40.00%	55.56%	27.27%
5	176088956	176851727	5q35.2-q35.3	9	6	3	45.00%	66.67%	27.27%
5	176946793	179205640	5q35.3	8	6	2	40.00%	66.67%	18.18%
8	47703628	48686513	8q11.1-q11.21	6	6	0	30.00%	66.67%	0.00%
8	85579256	93417060	8q21.2-q22.1	6	4	2	30.00%	44.44%	18.18%
8	109774071	110527427	8q23.1	6	4	2	30.00%	44.44%	18.18%
8	113540571	115896284	8q23.3	7	5	2	35.00%	55.56%	18.18%
8	117782567	117927576	8q24.11	7	5	2	35.00%	55.56%	18.18%
8	123323854	124468697	8q24.13	8	6	2	40.00%	66.67%	18.18%
8	128130585	131754948	8q24.21-q24.22	9	7	2	45.00%	77.78%	18.18%
8	137882907	140116037	8q24.23-q24.3	7	5	2	35.00%	55.56%	18.18%
8	145785956	146005188	8q24.3	6	5	1	30.00%	55.56%	9.09%
9	163131	38571787	9p24.3-p13.2	8	3	5	40.00%	33.33%	45.45%
9	71889510	141122055	9q21.13-q34.3	12	7	5	60.00%	77.78%	45.45%
14	106410478	106512963	14q32.33	6	5	1	30.00%	55.56%	9.09%
20	70580	4168304	20p13	9	5	4	45.00%	55.56%	36.36%
20	29833609	34687137	20q11.21-q11.23	17	9	8	85.00%	100.00%	72.73%
20	36444066	37986609	20q11.23-q12	16	8	8	80.00%	88.89%	72.73%
20	38430006	40351587	20q12	16	8	8	80.00%	88.89%	72.73%
20	41617589	42924016	20q13.12	16	8	8	80.00%	88.89%	72.73%
20	51151491	53396641	20q13.2	17	9	8	85.00%	100.00%	72.73%
20	61153381	61863831	20q13.33	14	7	7	70.00%	77.78%	63.64%
20	62208157	62654677	20q13.33	14	8	6	70.00%	88.89%	54.55%

LOSSES									
Chromosome	Start position	End position	Cytoband	Samples (n=)	Crisis (n=)	No crisis (n=)	% Total	% Crisis	% No crisis
2	97526630	97547543	2q11.2	8	4	4	40.00%	44.44%	36.36%
2	132307267	169457945	2q21.2-q24.3	8	4	4	40.00%	44.44%	36.36%
2	239000632	239063582	2q37.3	9	5	4	45.00%	55.56%	36.36%
3	64066	11848995	3p26.3-p25.2	13	6	7	65.00%	66.67%	63.64%
3	60445746	90282056	3p14.2-p11.1	15	7	8	75.00%	77.78%	72.73%
4	3475992	3535404	4p16.3	12	4	8	60.00%	44.44%	72.73%
4	45518913	49083231	4p13-p11	6	4	2	30.00%	44.44%	18.18%
4	148601450	156726238	4q31.22-q32.1	6	3	3	30.00%	33.33%	27.27%
8	360369	22021010	8p23.3-p21.3	6	5	1	30.00%	55.56%	9.09%
8	39237438	39380595	8p11.23	6	5	1	30.00%	55.56%	9.09%
12	163593	38497380	12p13.33-q12	6	3	3	30.00%	33.33%	27.27%
12	38503456	53699602	12q12-q13.2	6	3	3	30.00%	33.33%	27.27%
12	61177213	85549706	12q14.1-q21.32	6	3	3	30.00%	33.33%	27.27%
12	121285002	133779017	12q24.31-q24.33	7	3	4	35.00%	33.33%	36.36%
13	34725259	35770047	13q13.3	11	5	6	55.00%	55.56%	54.55%
16	96766	1404007	16p13.3	8	4	4	40.00%	44.44%	36.36%
16	29815121	29829346	16p11.2	6	3	3	30.00%	33.33%	27.27%
16	78424175	78609668	16q23.1-q23.2	8	5	3	40.00%	55.56%	27.27%
18	5988578	14978075	18p11.31-p11.21	7	5	2	35.00%	55.56%	18.18%
18	77158676	77307340	18q23	7	4	3	35.00%	44.44%	27.27%
18	77628089	77670636	18q23	6	3	3	30.00%	33.33%	27.27%
22	16053473	17599429	22q11.1-q11.21	8	5	3	40.00%	55.56%	27.27%
22	20293142	20306772	22q11.21	10	6	4	50.00%	66.67%	36.36%
22	24341374	24406990	22q12.1	8	4	4	40.00%	44.44%	36.36%
22	38470042	38507492	22q13.1	17	8	9	85.00%	88.89%	81.82%
22	50613880	51218950	22q13.33	17	8	9	85.00%	88.89%	81.82%

To determine whether specific chromosomal aberrations could potentially contribute to the outgrowth of immortal cells during crisis, we compared the chromosomal profiles of immortal descendants of cell lines with (n=9) and without (n=11) crisis. Gains of two regions in chromosome 5p, 3 regions on 8q and a region on 9q as well as loss of two regions on chromosome 8p were observed significantly (χ^2^ test, FDR<0.10) more often in immortal cells that underwent crisis, whereas loss of a region on chromosome 4p was significantly (χ^2^ test, FDR<0.10) more frequent in immortal cells showing continuous growth without an apparent crisis period (Table 2).

**Table 2:** Regions found to be statistically differentially altered in immortal cells with and without preceding crisis

					Crisis		No crisis	
Chromosome	Start position	End position	Cytoband	FDR	% Gains	% Losses	% Gains	% Losses
4	3560478	3872321	4p16.3	0.074	0.0	33.3	0.0	72.7
5	22149	25639915	5p15.33-p14.1	0.059	55.6	0.0	0.0	0.0
5	29048764	45972937	5p13.3-p11	0.045	63.0	0.0	0.0	3.0
7	54185	920862	7p22.3	0.088	33.3	22.2	0.0	9.1
7	1286655	5273452	7p22.3-p22.1	0.059	38.9	24.1	0.0	4.5
8	32556510	39222367	8p12-p11.23	0.059	0.0	55.6	0.0	0.0
8	39416498	42697785	8p11.23-p11.21	0.059	0.0	55.6	0.0	0.0
8	47471880	48916655	8q11.1-q11.21	0.060	59.3	3.7	0.0	9.1
8	128130585	129599581	8q24.21	0.074	77.8	0.0	18.2	0.0
8	137882907	139393884	8q24.23	0.095	66.7	0.0	18.2	0.0
9	138374663	140343467	9q34.3	0.074	77.8	22.2	45.5	0.0
11	65318368	65418337	11q13.1	0.038	38.9	27.8	0.0	0.0
11	66046441	66101855	11q13.2	0.059	33.3	22.2	0.0	0.0
11	67175527	94111514	11q13.2-q21	0.056	38.9	18.5	0.0	0.0
23	2741293	4764096	23p22.33-p22.32	0.059	38.9	16.7	0.0	0.0
23	19904414	21962316	23p22.12-p22.11	0.059	33.3	22.2	0.0	0.0
23	154631204	154929220	23q28	0.053	38.9	38.9	0.0	18.2

FDR- False discovery rate

Interestingly, the gains at 5p13.3-p11 and 5p15.33-p14.1, which were only observed in immortalized cells with crisis, contain the DROSHA and hTERT gene, respectively. Detectable hTERT mRNA expression, as determined in our previous study [14], coincided with occurrence of the 5p copy number gain in these cells. Conversely, in cells without crisis hTERT expression was detectable at earlier passages and these cells did not acquire the 5p gain (see Supplementary Table 1). In addition, gains more frequently observed in immortalized cells with crisis compared to those without crisis also affected well-known oncogenes MYC (8q24.21) and CCND1 (11q13.3). Low level amplifications of hTERT, DROSHA, MYC and CCND1 have been implicated in cervical carcinogenesis before [17–19]. An overview of all genes located in the differentially altered regions between immortal cells with and without crisis is given in Supplementary Table 2.

Together, these results suggest that the increased number of chromosomal aberrations, and in particular chromosomal gains, in cells that underwent a crisis period may have contributed to the acquisition of an immortal phenotype.

### Chromothripsis in HPV16-immortalized cells resulted in a gain of the oncogene MYC

**Figure 5:**
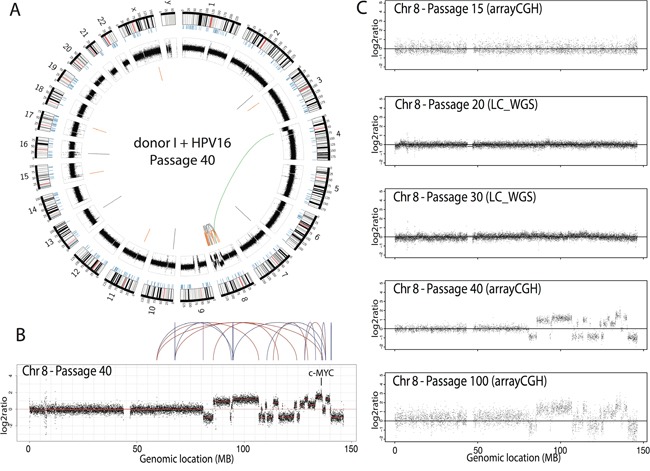
Cell line with chromothripsis **A.** Circos plot of a single cell line (donor I, HPV16, passage 40) showing the interchromosomal translocations (orange), inversions (grey) and intrachromosomal translocations (green). **B.** Chromothripsis on the q-arm of chromosome 8. **C.** DNA copy number profiles of chromosome 8 over 5 passages of the cell line presented with chromothripsis, either assessed with arrayCGH or low-coverage, whole genome sequencing (LC-WGS).

One cell line (immortal cell line of donor I containing HPV16, no crisis, passage 40) showed relatively few CNA genome-wide except for chromosome 8, with a strikingly high number of clustered breaks. Analysis after long-range paired-end sequencing revealed high number of intrachromosomal rearrangements on chromosome 8 and a single translocation connecting chromosome 4 with chromosome 8 (Figure 5A). Interestingly, the highest peak was situated at 8q24.21, the region encoding the oncogene MYC (Figure 5B). Such localized massive genomic rearrangement is compatible with “chromothripsis”, in which shattering of several genomic regions is hypothesized to occur during a single catastrophic event [20]. Chromothripsis was neither evident in pre-immortal cells (passage 15 cells; arrayCGH results), nor in cells at passage 20 and passage 30 (low-coverage sequencing results), but was retained in late immortal cells (passage 100; array CGH results) (Figure 5C). To the best of our knowledge, this is the first cell line with chromothripsis that is established *in vitro* and that exhibits an immortal, but non-transformed phenotype (unable to grow anchorage independent/data not shown) and represents a precancerous lesion when cultured on organotypic rafts [14].

## DISCUSSION

In the present study we showed a progressive increase in chromosomal aberrations during hrHPV-mediated immortalization. Interestingly, we found that the frequency of aberrations was inversely related to the immortalization capacity of the ten hrHPV types analyzed. Cell lines immortalized by HPV types with a higher immortalization capacity, including HPV16, 18, 31, 33 and 35, showed significantly fewer CNA compared to the cell lines immortalized by HPV types with a lower immortalization capacity (i.e. HPV45, 51, 59, 66 and 70), in which immortalization was preceded by a crisis period. The increased number of CNA in cells that encountered a crisis period was not linked to a differential induction of DNA double strand breaks, as determined by γ-H2A.X staining, between HPV types with low and high immortalization capacities.

In all hrHPV-transduced HFKs a significant increase in DNA-double strand breaks (γ-H2A.X positive cells) compared to controls was observed, with most cells being tested at early passage. Yet, most CNA became apparent at later passages following immortalization. This observation is in line with a previous report by Bester et al. in which DNA damage was immediately induced following expression of HPVE6 and E7 due to forced entry into S-phase without concomitant up-regulation of deoxyribonucleoside triphosphate synthesis, whereas loss of heterozygosity and copy number variations became evident after 100-250 population doublings [21]. Since in the context of full length HPV the early genes E1 and E2 are also known to contribute to DNA damage induction upon HPV infection or during transformation [22, 23], the DNA damage and genomic instability observed upon ectopic expression of E6 and E7 only may be an underestimation of the natural process.

Cell lines without a crisis period and relatively few CNA were previously shown to express hTERT at early passages and to more efficiently degrade p53, indicating that the respective E6 proteins are more oncogenic than the E6 proteins of HPV types with reduced immortalization capacity [14]. This suggests that earlier activation of telomerase upon hTERT upregulation mediated by oncogenic E6 activity [7, 24], reduces genomic instability by maintaining telomere function and suppression of chromosome fusions after chromosome breakage [25]. Accordingly, less oncogenic HPV types, which did not induce an early upregulation of hTERT [14], most likely require more CNA to induce telomerase and to enable a subset of cells with a genetic profile permissive for proliferation to emerge from crisis. Interestingly, regions on chromosome 5p, where the hTERT and DROSHA gene are located, were exclusively gained in the majority of cell lines that encountered a crisis and not in cell lines that showed a continuous growth (Figure 4). In fact, upregulation of hTERT expression coincided with the detection of a 5p gain in the immortal cells that underwent crisis. This suggests that a 5p gain resulting in increased hTERT expression may have facilitated immortalization. Although a gain of the hTERT locus is a common feature in cervical cancer [26, 27], it is unknown whether this is type dependent. Similarly, increased expression of DROSHA related to a copy number gain was described before in cervical cancer and was shown to result in altered miRNA expression profiles [17].

Regions on chromosomes 8q and 9q were more often gained in cell lines with a crisis period and may harbor (putative) oncogenes that provide a growth advantage in cells infected with less carcinogenic virus types during crisis. The lost region on chromosome 4p was significantly more frequent in cells that showed a continuous growth. Loss of heterozygosity at this region has been demonstrated in cervical carcinomas [28].

Overall, gain of chromosome 20q and loss of chromosome 3p and 22 represented the most frequent events in the HPV-immortalized cell lines [29]. A gain at chromosome 20q has previously been described to be essential for HPV-induced immortalization [29–31] and to be associated with HPV16 E7 induced inactivation of the pRB pathway [32]. Loss of chromosome 3p, particularly at position 3p12-14, has been recognized as one of the most common events in cervical squamous cell carcinoma [13, 33]. CNA at chromosome 22 are uncommon in HPV-associated lesions, though breakage-fusion translocations involving chromosome 22 have been described in HeLa cells [34]. Frequent CNA at chromosome 22 as detected i present study were also found in other *in vitro* transformed HPV-containing cell lines and may represent an effect of *in vitro* culturing [9, 35, 36].

Interestingly and to the best of our knowledge, we are the first to report an *in vitro* generated HPV-immortalized cell line with chromothripsis. Chromothripsis is characterized by massive rearrangements involving one or a few chromosomes and is a rare event that has been observed in 2 to 3% of all cancers [20]. In one of our HPV16-immortalized cell lines we observed chromothripsis at passage 40. No chromothripsis was observed at earlier passages, i.e. passages 15, 20 and 30. This supports the notion that chromothripsis results from a single catastrophic event that either occurred after passage 30 or remained undetectable until passage 40. High level intrachromosomal rearrangements occurred in chromosome 8, and resulted in amplification of the oncogene MYC. Since MYC amplification was only detected at passage 40, which is approximately 20 passages after immortalization, the biological advantage remains to be determined. Activation of MYC as a result of viral integration at the MYC locus has been recognized as a potential mechanism of HPV-induced carcinogenesis [37]. Current data indicate that activation of MYC as a result of chromothripsis or CNA may represent an alternative mechanism contributing to HPV-induced carcinogenesis. Given the fact that these HPV-immortalized cell lines resemble precancerous lesions of the cervix upon growth in three-dimensional organic raft cultures [14], these data indicate that chromothripsis may represent an early event already occurring in precancerous lesions.

Our preliminary data on mutation analysis of 48 cancer-related genes (TruSeq Amplicon panel Illumina) indicate that somatic mutations are extremely rare in HPV-immortalized cells. Only one mutation in GNAQ was found in one cell line. Furthermore, previous DNA methylation analysis of 15 genes in HPV16-, 18-, 31-, 33-, 45-, 66- and 70- immortalized cells showed no major differences in methylation patterns between the various HPV-immortalized cell lines [15]. Apparently, the HPV-type dependent immortalization capacity, as reflected by different growth behaviors of HPV-transduced HFKs, is particularly associated with variability in copy number aberrations, including those associated with gains, and deletions that may affect expression levels of the associated driver proteins. This suggests that the CNA as described in this study are likely to provide a growth advantage and enable the bypass of crisis.

In conclusion, analysis of HPV-immortalized HFK cell lines transduced with ten different HPV types revealed that the induction of DNA-double strand breaks (phospho-histone H2A.X induction) is HPV-type independent. Conversely, the extent of CNA in immortal cells appeared to be HPV-type dependent. The percentage of CNA was found to be inversely related to the immortalization capacity of the virus type present. In particular the number of chromosomal gains was significantly increased in HPV-immortalized cells that underwent a crisis period compared to HPV-immortalized cells that grew continuously.

The reduced *in vitro* immortalization capacity and apparent requirement of more genetic host cell aberrations to support an unlimited lifespan of HPV types 45, 51, 59, 66 and 70, compared to HPV types 16, 18, 31, 33 and 35, may in part explain their mostly lower prevalence in cervical cancers and precursor lesions.

## MATERIALS AND METHODS

### Cell culture and DNA isolation

Cell lines were retrieved and cultured as described before [14]. Briefly, HFKs were isolated from the foreskins of three independent donors as described previously [6]. After two initial passages, HFKs were transduced with amphotropic retroviruses expressing the E6/E7 open reading frame of HPV types 16, 18, 31, 33, 35, 45, 51, 59, 66 and 70. Geneticin (Life Technology, Breda, The Netherlands) selection was performed 48 h after transduction. All cell lines were cultured in defined keratinocyte serum-free medium (SFM) (Life Technologies, Breda, The Netherlands) containing 5ng/ml EGF and 50ng/ml bovine pituitary extract, 100 U/mL penicillin, 100 μg/mL streptomycin, 2 mmol/L L-glutamine (Life Technologies) and 80 μg/ml geneticin (Life Technologies) at 37°C and 5% CO_2_. Splitting dates and dilutions were noted to determine proliferation characteristics. Exponentially growing cells were harvested by trypsinization. Genomic DNA was isolated from cell pellets by proteinase K digestion followed by UltraPure™ Phenol:Chloroform:Isoamyl Alcohol (Life Technologies) extraction as described previously [38].

An overview of the various cell lines and passages used in the different experiments is given in Supplementary Table 1.

### Phospho-histone H2A.X (γ-H2A.X) immunofluorescence staining

Cells were grown in chamber slides (Nunc), washed with PBS and pre-permeabilized with 0.25% Triton X-100 in PBS (1 min on ice). Cells were fixed with 4% paraformaldehyde/PBS (15 min at room temperature) and permeabilized with 0.5% Triton X-100 in PBS (10–20 min at room temperature). Unspecific binding sites were blocked with 10% BSA/PBS for 1 h at room temperature. Slides were incubated for 2 h at room temperature with anti-phospho-Histone H2A.X (Millipore, clone JBW301, 1:1000) in blocking buffer and excess antibody was removed by four washing steps with 0.2% Triton X-100/PBS. Slides were then incubated with secondary antibody labeled with Alexa 488 (Invitrogen, 1:1000) and DAPI (1:1000) for 1 h at room temperature. Finally, slides were washed four times with 0.2% Triton X-100/PBS and embedded in vectashield (Vector laboratories, UK). Slides were analyzed with a fluorescent microscope (DM5000, Leica). Experiments were performed in duplicate and the percentage of phospho-histone H2A.X positive cells was scored from digitized images using constant light intensities. Approximately, 100-200 cells per experiment were counted and scored for γ-H2A.X positivity.

### ArrayCGH analysis and pre-processing

Labeling and hybridization to the 2×105K arrayCGH platform (Agilent Technologies, Palo Alto, USA) was performed as described previously [29]. Downstream analysis was performed and plots were made using the statistical programming language R version 3.0.1. Log2-ratios of signal intensities between sample and reference for every probe were median-normalized and post-segmentation mode normalization was performed [39]. The arrayCGH data has been made available through the Gene Expression Omnibus (GEO;http://www.ncbi.nlm.nih.gov/projects/geo/) through series accession number GSE72063.

### Paired-end and mate-pair sequencing and structural variant detection

Whole genome low-coverage short insert size library preparation was performed using the NEBNext Ultra DNA Library Prep Kit (New England Biolabs) and long-range (4422 bp insert size) mate-pair library preparation was done using the Nextera Mate Pair Sample Prep Kit (Illumina Inc.), both according to the manufacturer's protocol. Sequencing was performed on Illumina HiSeq instruments to an average 3x spanning coverage for short insert size paired-end and 38x spanning coverage for mate-pair, with the raw length of the reads displaying a median of 101bp. Structural variants larger than 5,000 bp were detected using the DELLY tool [40], as previously described [41]. DNA copy number analysis was performed using qDNAseq [42]. The sequencing data has been made available through the European Nucleotide Archive (ENA; http://www.ebi.ac.uk/ena; dataset ID: PRJEB9176.)

### Statistical analysis

The χ^2^-test as available in the R-package CGHtest was used to determine possible differences between chromosomal profiles of cell lines with a high immortalization capacity and cell lines with a low immortalization capacity [43]. The test includes a permutation-based false discovery rate (FDR) correction for multiple testing. Differences were considered to be significant if the FDR was <0.10.

## SUPPLEMENTARY TABLES




