# Supplementary material for: Immortalization capacity of HPV types is inversely related to chromosomal instability
Source: Oncotarget. 2016 Mar 14;7(25):37608–21. doi: 10.18632/oncotarget.8058 (PMC5122336; doi:10.18632/oncotarget.8058)
Supplement: Supplementary file 1 [file oncotarget-07-37608-s001.pdf]

Immortalization capacity of HPV types is inversely related to chromosomal instability

SUPPLEMENTARY TABLES

Supplementary Table 1: Overview of HPV-transduced cell lines and passages used for γ-H2AX staining and arrayCGH, presence of 5p gain and onset of hTERT mRNA expression

Donor	HPV type	crisis	yH2Ax (passage number)	arrayCGH mortal (passage number)	Array CGH immortal	5p gain in immortal cells	Onset hTERT mRNA expression*
I	16	NO	p16	p15	p40	NO	Early
	18	NO	p20	p17	p45	NO	Early
	31	NO	p27	p16	p45	NO	Early
	33	NO	p21	p17	p46	NO	Early
	35	NO	p18	p15	p45	NO	Late
	45	YES	p19	p25	p45	YES	Late
	51	YES	p13	p15	p46	YES	Late
	59	YES	p18	p17			
	66	YES	p34	p12	p45	NO	Late
	70	YES	p12	p15	p45	NO	Middle(p15)
II	16	NO		ND	p45	NO	Early
	18	NO		ND	p45	NO	-
	31	NO		ND	p40	NO	Late
	35	NO		ND	p45	NO	Early
	45	YES-no immortalization		p20			
	51	YES-no immortalization		p14			
	59	YES		p15	p45	YES	Early
	66	YES		p13	p45	YES	Late
	70	YES		p15	p45	YES	Late
III	33	NO		ND	p45	NO	Early
	35	NO		ND	p45	NO	Late
	45	YES-no immortalization		p19			
	51	YES-no immortalization		p15			
	59	YES		p15	p45	NO	Early
	70	YES		p14	p45	YES	Late

*Early:passage 7-10; Late: passage ≥25

Supplementary Table 2: Overview of all genes located in the differentially altered regions between immortal cells with and without crisis

See Supplementary File 1
