# Supplementary material for: Immortalization capacity of HPV types is inversely related to chromosomal instability
Source: Oncotarget. 2016 Mar 14;7(25):37608–21. doi: 10.18632/oncotarget.8058 (PMC5122336; doi:10.18632/oncotarget.8058)
Supplement: Supplementary file 2 [file oncotarget-07-37608-s002.docx]

| **Supplementary Table 2:** Overview of all genes located in the differentially altered regions between immortal cells with and without crisis | | | | |
| --- | --- | --- | --- | --- |
| **Chromosome** | Start (bp) | End (bp) | Cytoband | Refseq genes (hg18) |
| **4** | 3560478 | 3872321 | 4p16.3 | LINC00955; LOC100133461; ADRA2C |
| **5** | 22149 | 25639915 | 5p15.33-p14.1 | PLEKHG4B; LRRC14B; CCDC127; SDHA; HRAT5; PDCD6; AHRR; EXOC3-AS1; EXOC3; PP7080; SLC9A3; LOC100288152; MIR4456; LOC100996325; CEP72; TPPP; ZDHHC11; BRD9; TRIP13; LOC100506688; NKD2; SLC12A7; MIR4635; CTD-3080P12.3; SLC6A19; SLC6A18; **TERT**; MIR4457; CLPTM1L; LINC01511; SLC6A3; LPCAT1; MIR6075; SDHAP3; LOC728613; MIR4277; MRPL36; NDUFS6; LOC101929034; IRX4; CTD-2194D22.4; LOC100506858; IRX2; C5orf38; LINC01377; LINC01019; LINC01017; IRX1; LOC101929153; LINC01020; CTD-2297D10.2; ADAMTS16; ICE1; FLJ33360; MED10; UBE2QL1; LINC01018; NSUN2; SRD5A1; LOC100505625; PAPD7; MIR4278; MIR4454; LOC442132; ADCY2; C5orf49; FASTKD3; MTRR; LOC729506; MIR4458HG; MIR4458; LOC101929284; SEMA5A; MIR4636; CTD-2201E9.1; SNHG18; SNORD123; TAS2R1; LOC285692; FAM173B; CCT5; CMBL; MARCH6; ROPN1L-AS1; ROPN1L; MIR6131; LOC101929412; ANKRD33B; DAP; CTNND2; LINC01194; DNAH5; TRIO; FAM105A; OTULIN; ANKH; LOC100130744; MIR4637; LOC101929454; FBXL7; CTD-2350J17.1; MIR887; MARCH11; LOC101929505; ZNF622; FAM134B; LOC101929524; MYO10; LOC285696; BASP1; LOC401177; CDH18; GUSBP1; CDH12; PMCHL1; PRDM9; C5orf17; CDH10; LOC340107 |
| **5** | 29048764 | 45972937 | 5p13.3-p11 | LOC101929645; LOC101929660; LOC101929681; CDH6; **DROSHA**; C5orf22; PDZD2; MIR4279; GOLPH3; MTMR12; ZFR; MIR579; SUB1; NPR3; LOC340113; TARS; ADAMTS12; RXFP3; SLC45A2; C1QTNF3-AMACR; AMACR; C1QTNF3; RAI14; MIR7641-2; TTC23L; RAD1; BRIX1; DNAJC21; AGXT2; PRLR; SPEF2; IL7R; CAPSL; LOC100506406; UGT3A1; UGT3A2; LMBRD2; MIR580; SKP2; NADK2; RANBP3L; SLC1A3; NIPBL-AS1; NIPBL; C5orf42; NUP155; WDR70; GDNF; GDNF-AS1; EGFLAM; EGFLAM-AS4; EGFLAM-AS2; LIFR; LIFR-AS1; MIR3650; OSMR-AS1; LINC01265; OSMR; RICTOR; FYB; C9; DAB2; LOC101926940; LINC00603; PTGER4; TTC33; PRKAA1; LOC100506548; RPL37; SNORD72; CARD6; C7; MROH2B; C6; PLCXD3; OXCT1; OXCT1-AS1; C5orf51; FBXO4; LOC101926960; GHR; CCDC152; SEPP1; FLJ32255; LOC648987; ANXA2R; LOC153684; LOC100132356; LOC100506639; ZNF131; NIM1K; HMGCS1; CCL28; C5orf28; C5orf34; PAIP1; NNT-AS1; NNT; FGF10; FGF10-AS1; BRCAT107; BRCAT54; MRPS30; HCN1 |
| **7** | 54185 | 920862 | 7p22.3 | LOC100507642; FAM20C; WI2-2373I1.2; LOC442497; PDGFA; HRAT92; PRKAR1B; LOC101926963; DNAAF5; SUN1; GET4 |
| **7** | 1286655 | 5273452 | 7p22.3-p22.1 | MICALL2; INTS1; MAFK; TMEM184A; PSMG3; PSMG3-AS1; TFAMP1; ELFN1; ELFN1-AS1; MAD1L1; MIR4655; FTSJ2; NUDT1; SNX8; MIR6836; EIF3B; CHST12; LOC101927181; GRIFIN; LFNG; MIR4648; BRAT1; IQCE; TTYH3; AMZ1; GNA12; CARD11; LOC100129603; SDK1; FOXK1; AP5Z1; MIR4656; RADIL; PAPOLB; MMD2; RNF216P1; RBAK; RBAK-RBAKDN; RBAKDN; ZNF890P; WIPI2 |
| **8** | 32556510 | 39222367 | 8p12-p11.23 | NRG1; FUT10; MAK16; TTI2; RNF122; DUSP26; LINC01288; UNC5D; LOC101929550; KCNU1; MIR1268A; LINC01605; ZNF703; LOC101929622; LOC102723701; ERLIN2; LOC728024; PROSC; ADGRA2; BRF2; RAB11FIP1; GOT1L1; ADRB3; EIF4EBP1; ASH2L; STAR; LSM1; BAG4; DDHD2; PPAPDC1B; WHSC1L1; LETM2; FGFR1; C8orf86; RNF5P1; TACC1; PLEKHA2; HTRA4; TM2D2; ADAM9; ADAM32; ADAM5 |
| **8** | 39416498 | 42697785 | 8p11.23-p11.21 | LOC100130964; ADAM18; ADAM2; IDO1; IDO2; C8orf4; ZMAT4; SFRP1; GOLGA7; GINS4; LOC102723729; GPAT4; NKX6-3; ANK1; MIR486-1; MIR486-2; KAT6A; AP3M2; PLAT; LOC101929897; IKBKB; POLB; DKK4; VDAC3; SLC20A2; SMIM19; CHRNB3; CHRNA6; THAP1 |
| **8** | 47471880 | 48916655 | 8q11.1-q11.21 | LINC00293; LOC100287846; SPIDR; CEBPD; PRKDC; MCM4 |
| **8** | 128130585 | 129599581 | 8q24.21 | CASC19; CCAT1; CASC21; CASC8; CCAT2; POU5F1B; CASC11; **MYC**; MIR1204; PVT1; TMEM75; MIR1205; MIR1206; MIR1207; MIR1208; LINC00824 |
| **8** | 137882907 | 139393884 | 8q24.23 | LOC101927915; FAM135B |
| **9** | 138374663 | 140343467 | 9q34.3 | PPP1R26; C9orf116; MRPS2; LOC101928525; LCN1; OBP2A; PAEP; LINC01502; GLT6D1; LCN9; SOHLH1; KCNT1; CAMSAP1; UBAC1; NACC2; C9orf69; LHX3; QSOX2; DKFZP434A062; GPSM1; DNLZ; CARD9; SNAPC4; SDCCAG3; PMPCA; INPP5E; SEC16A; C9orf163; NOTCH1; MIR4673; MIR4674; LINC01573; EGFL7; MIR126; AGPAT2; FAM69B; SNHG7; SNORA43; SNORA17; LCN10; LCN6; LOC100128593; MIR6722; LCN8; LCN15; TMEM141; CCDC183; CCDC183-AS1; RABL6; MIR4292; C9orf172; PHPT1; MAMDC4; EDF1; TRAF2; MIR4479; FBXW5; C8G; LCN12; PTGDS; LCNL1; C9orf142; CLIC3; ABCA2; C9orf139; FUT7; NPDC1; ENTPD2; SAPCD2; UAP1L1; MAN1B1-AS1; MAN1B1; DPP7; GRIN1; LRRC26; MIR3621; TMEM210; ANAPC2; SSNA1; TPRN; TMEM203; NDOR1; RNF208; CYSRT1; RNF224; SLC34A3; TUBB4B; FAM166A; C9orf173-AS1; C9orf173; NELFB; TOR4A; NRARP; EXD3; NOXA1; ENTPD8; NSMF |
| **11** | 65318368 | 65418337 | 11q13.1 | LTBP3; SSSCA1-AS1; SSSCA1; FAM89B; EHBP1L1; KCNK7; MAP3K11; PCNXL3; MIR4690; SIPA1; MIR4489 |
| **11** | 66046441 | 66101855 | 11q13.2 | CNIH2; YIF1A; TMEM151A; CD248; RIN1 |
| **11** | 67175527 | 94111514 | 11q13.2-q21 | TBC1D10C; CARNS1; RPS6KB2; PTPRCAP; CORO1B; GPR152; CABP4; TMEM134; AIP; MIR6752; PITPNM1; CDK2AP2; CABP2; GSTP1; C11orf72; NDUFV1; DOC2GP; NUDT8; TBX10; ACY3; ALDH3B2; FAM86C2P; UNC93B1; ALDH3B1; NDUFS8; MIR7113; MIR4691; TCIRG1; MIR6753; CHKA; SUV420H1; C11orf24; LRP5; PPP6R3; GAL; MTL5; CPT1A; MRPL21; IGHMBP2; MRGPRD; MRGPRF; MRGPRF-AS1; TPCN2; MIR3164; LOC338694; MYEOV; LINC01488; **CCND1**; ORAOV1; FGF19; FGF4; FGF3; LOC101928443; ANO1-AS2; ANO1; FADD; PPFIA1; MIR548K; CTTN; SHANK2; SHANK2-AS1; SHANK2-AS3; MIR3664; FLJ42102; DHCR7; NADSYN1; MIR6754; KRTAP5-7; KRTAP5-8; KRTAP5-9; KRTAP5-10; KRTAP5-11; FAM86C1; ALG1L9P; ZNF705E; DEFB108B; LOC100133315; LOC100129216; RNF121; IL18BP; NUMA1; LOC100128494; MIR3165; LRTOMT; LAMTOR1; ANAPC15; FOLR3; FOLR1; FOLR2; INPPL1; PHOX2A; CLPB; LINC01537; PDE2A; MIR139; ARAP1; STARD10; MIR4692; ATG16L2; FCHSD2; MIR4459; P2RY2; P2RY6; ARHGEF17; RELT; FAM168A; PLEKHB1; RAB6A; MRPL48; COA4; PAAF1; DNAJB13; UCP2; UCP3; C2CD3; PPME1; P4HA3; LOC101928580; PGM2L1; KCNE3; LIPT2; POLD3; CHRDL2; MIR4696; RNF169; XRRA1; SPCS2; NEU3; OR2AT4; SLCO2B1; TPBGL; ARRB1; MIR326; RPS3; SNORD15A; SNORD15B; KLHL35; GDPD5; SERPINH1; MAP6; MOGAT2; LOC283214; DGAT2; UVRAG; WNT11; PRKRIR; LOC100506127; C11orf30; LRRC32; GUCY2EP; TSKU; LOC101928837; ACER3; B3GNT6; CAPN5; OMP; MYO7A; GDPD4; PAK1; LOC646029; AQP11; CLNS1A; RSF1; AAMDC; INTS4; NDUFC2-KCTD14; KCTD14; RNU6-83P; THRSP; NDUFC2; ALG8; KCTD21-AS1; KCTD21; USP35; GAB2; LOC101928865; NARS2; LOC101928896; TENM4; MIR708; MIR5579; LOC101928944; LOC101928989; MIR4300; FAM181B; PRCP; DDIAS; RAB30; SNORA70E; RAB30-AS1; PCF11; ANKRD42; CCDC90B; DLG2; TMEM126B; TMEM126A; CREBZF; CCDC89; SYTL2; CCDC83; PICALM; EED; MIR6755; C11orf73; CCDC81; ME3; PRSS23; OR7E2P; FZD4; LOC100506368; TMEM135; RAB38; MIR3166; CTSC; GRM5; GRM5-AS1; TYR; NOX4; FOLH1B; TRIM77; TRIM49; TRIM53AP; TRIM64B; TRIM49D2; TRIM49D1; TRIM64; TRIM49C; UBTFL1; NAALAD2; CHORDC1; DISC1FP1; MIR4490; MIR1261; FAT3; MTNR1B; SLC36A4; CCDC67; SMCO4; CEP295; SCARNA9; SNORA25; SNORA32; SNORD6; SNORA1; SNORA8; SNORD5; SNORA18; MIR1304; SNORA40; TAF1D; C11orf54; MED17; VSTM5; HEPHL1; PANX1; IZUMO1R; GPR83 |
| **23** | 2741293 | 4764096 | 23p22.33-p22.32 | SHOX; CRLF2; CSF2RA; MIR3690; IL3RA; SLC25A6; LINC00106; ASMTL-AS1; ASMTL; P2RY8; AKAP17A; ASMT; DHRSX; ZBED1; MIR6089; CD99P1; LINC00102; CD99; XG; XGY2 |
| **23** | 19904414 | 21962316 | 23p22.12-p22.11 | SH3KBP1; CXorf23; LOC729609; MAP7D2; MIR23C; EIF1AX; SCARNA9L; EIF1AX-AS1; RPS6KA3; CNKSR2; KLHL34; SMPX; MBTPS2; YY2; SMS |
| **23** | 154631204 | 154929220 | 23q28 | F8A1; F8A2; F8A3; MIR1184-2; MIR1184-1; MIR1184-3; H2AFB2; H2AFB3; H2AFB1; TMLHE-AS1; LOC101927830; TMLHE; SPRY3 |
| Genes of interest were highlighted in bold and red | | | | |
